# Haart impact on prevalence of chronic otitis media in Brazilian HIV-infected children

**DOI:** 10.1016/S1808-8694(15)30997-6

**Published:** 2015-10-19

**Authors:** Raimar Weber, Carlos Diógenes Pinheiro Neto, Ivan Dieb Miziara, Bernardo Cunha Araújo Filho

**Affiliations:** a MD, Otorhinolaryngologist Resident at the Department of Otorhinolaryngology and Ophthalmology of the Medical School of the University of São Paulo; b Assistant Professor - Department of Otorhinolaryngology and Ophthalmology of the Medical School of the University of São Paulo; c MD, Otorhinolaryngologist, Postgraduate Student - Department of Otorhinolaryngology and Ophthalmology of the Medical School of the University of São Paulo. Study carried out at the Department of Otorhinolaryngology and Ophthalmology of the Medical School of the University of São Paulo

**Keywords:** haart, hiv infections, chronic otitis media

## Abstract

The advent of new antiretroviral drugs such as protease inhibitors has generated sensible changes in morbity and mortality in HIV-infected patients.

**Objectives:**

To evaluate the impact of Highly Active Antiretroviral Therapy (HAART) on the prevalence of chronic otitis media in HIV-infected pediatric population.

**Methods:**

We analyzed medical charts of 471 children aged zero to 12 years and 11 months with HIV infection from an Ambulatory of ENT and AIDS. Children were divided according to the age: 0 to 5 years and 11 months and 6 to 12 years and 11 months and classified as having chronic otitis media based on history, physical examination, audiologic and tympanometric data. Prevalence of chronic otitis media, as well as CD4+ lymphocyte count were compared between groups in use of HAART and the group without HAART.

**Results:**

Out of 459 children, 65 (14.2%) had chronic otitis media. We observed that in children aged 0 to 5 years and 11 months who were taking HAART there was significant lower prevalence of chronic otitis media (p = 0.02). The use of HAART was associated to higher mean CD4+ lymphocyte count (p < 0.001).

**Conclusion:**

The use of HAART was associated to reduction in prevalence of chronic otitis media in HIV infected children, probably due to increase in mean CD4+ lymphocyte count.

## INTRODUCTION

The spread of HIV virus throughout the world has caused a terrible impact on childhood. It is estimated that 2.2 million children bellow 15 years of age are infected, according to data from UNAIDS/WHO of 2004^1^. In Brazil, until December of 2004, about 13,786 cases of children bellow 13 years of age had been registered by the Ministry of Health^2^. The discovery of new antiretroviral drugs, specially protease inhibitors have substantially reduced mortality and increased the life expectancy of these children^3^, ^4^. Such condition, associated to the fact that many of the initial signs and symptoms of HIV infections are otorhinolaryngological, has brought a greater number of these patients to be assessed by this medical specialty^3^, ^5^.

Otitis media (OM) is a common disease in childhood, and it is the most frequent acute disease seen by pediatricians^6^. OM prevalence in HIV-positive children may reach 80%^7^, and the seropositive child is more prone to have the disease (and in a higher degree of severity), when compared to immunecompetent children^8^. Moreover, for the HIV-immunocompromised child, the ENT should consider potential complications secondary to the disease, affecting the middle ear^9^, such as otomastoiditis and Central Nervous System involvement.

In 1987, Zidovudine was developed, a nucleoside inhibitor, analogous to the reverse transcriptase enzime (Reverse Transcriptase Nucleoside Inhibitors - RTNI), thus becoming the number one drug for the treatment of AIDS. Following that, non-nucleoside inhibitors of the same enzime (RTNNI) were developed, and in 1996, new antiretroviral drugs were introduced, represented by Protease Inhibitors (PI)^3^. The combination of these new drug classes (usually 2 RTNI + 1 PI or 1 RTNI + 1 RTNNI + 1 PI) started to be called Highly Active Antiretroviral Therapy - HAART).

Despite the high prevalence of OM in HIV-infected children, data related to a prevalence change in this disease in the pediatric population after the introduction of HAART remain scarce.

Thus, our major goal is to assess the changes associated to the use of HAART on the prevalence of chronic otitis media on a certain layer of Brazilian children infected by HIV.

## MATERIALS AND METHODS

We carried out a retrospective analysis on the charts of 471 children with prior diagnosis of HIV infection, with ages ranging between 0 and 12 years and 11 months, who were seen at the HIV/AIDS outpatient ward of the Otorhinolaryngology Department of the University Hospital of the Medical School of the University of São Paulo (HC-FMUSP), between January of 1990 and December of 2004.

12 children who were not receiving antiretroviral medication were taken off the sample. The 459 patients included in the study were receiving therapy with at least one antiretroviral drug for at least 5 months: 236 used the regimen without protease inhibitors, making up group ART (Antiretroviral Therapy – ART) and 223 were in HAART. The average time of treatment among those patients under ART was of 14 ± 4 months; and among patients using HAART was of 9 ± 2 months. Demographics (age, gender, place of birth and HIV infection via), as well as the antiretroviral regimens used by these 459 children are presented on Table 1. Data on the immunological classification of these children were also collected from the charts, based on the 1994 Center for Disease Control ‘Revised Classification System for Human Immunodeficiency Infection in Children Less than 13 Years of age’10, and are summarized on Table 2.

**Table 1:** Demographics and anti-retroviral therapies used by the 459 children infected.

Gender	

Male	258 (56,2%)
Female	201 (43,8%)

Age	

Average ± SD (years)	6,6 ± 2,5
0 to 5 years and 11 months	176 (38,3%)
6 to 12 years and 11 years	283 (61,7%)

HIV transmission means	

Vertical	387 (84,3%)
Blood Transfusion	18 (3,9%)
Unknown	54 (11,7%)

Origin	

Support institutions / hospitals	362 (79,0%)
Outpatient wards	97 (21,0%)

Anti-retroviral therapy	

2 ITRN + IP	133 (29,0%)
ITRN + ITRNN + IP	90 (19,6%)
AZT + DDI	45 (9,8%)
AZT + 3TC	82 (17,9%)
AZT only	90 (19,6%)
DDI only	19 (4,2%)


ITRN = Nucleoside Reverse Transcriptase Inhibitors: Zidovudine (AZT), Didanosine (DDI), Lamivudine (3TC); IP = Protease Inhibitors: Indinavir, Ritonavir, Saquinavir, Nelfinavir; ITRNN = Non-nucleoside Reverse Transcriptase Inhibitors: Nevirapine, Efavirenz.

**Table 2:** Distribution of the 459 children studied in the categories of immune classification, according to age range and HAART use or not.

	Age range			
	0 to 5 years and 11 months (n = 176)		6 to 12 years and 11 months (n = 283)	
Immune category	HAART (n = 70)	ART (n = 106)	HAART (n = 153)	ART (n = 130)
A1	12 (17,1%)	5 (4,7%)	54 (35,3%)	22 (16,9%)
A2	32 (45,7%)	21 (19,8%)	28 (18,3%)	13 (10,0%)
A3	1 (1,4%)	-	-	-
B1	1 (1,4%)	-	36 (23,5%)	24 (18,5%)
B2	19 (27,1%)	42 (39,6%)	24 (15,7%)	37 (28,5%)
B3	1 (1,4%)	3 (2,8%)	-	1 (0,8%)
C1	-	-	1 (0,7%)	-
C2	3 (4,3%)	26 (24,5%)	7 (4,5%)	26 (20,0%)
C3	1 (1,4%)	9 (8,5%)	3 (2,0%)	7 (5,4%)

On the first ambulatorial consultation we carried out a complete otorhinolaryngological exam, always by the same specialist (IDM). Table 3 depicts the otorhinolaryngological diagnosis of the 459 children studied. Otitis media was the most common otorhinolaryngological disease found (present in 33.1% of the children), and less than one-fourth (22.5%) of the children did not have any otorhinolaryngological diagnosis.

**Table 3:** Otorhinolaryngological diagnosis of the 459 Brazilian children infected with HIV.

Otorhinolaryngological Diagnosis	n (%)
Otitis Media	152 (33,1%)
Chronic	65 (14,2%)
Acute	48 (10,5%)
Secretory	39 (8,5%)
Cholesteatoma	1 (0,2%)
Otomastoiditis	1 (0,2%)
Oral Lesion †	144 (31,6%)
Neck lymphadenopathy	70 (15,3%)
Sinusitis	66 (14,4%)
Chronic	36 (7,8%)
Acute	30 (6,5%)
Adenoid hypertrophy	44 (9,6%)
Rhinitis	43 (9,4%)
Tonsillitis	37 (8,1%)
Peritonsillar abscess	3 (0,7%)
Laryngitis (acute)	3 (0,7%)
Kaposi Sarcoma	1 (0,2%)
None	103 (22,5%)

† Includes Parotid Enlargement

In order to classify and diagnose otitis media as acute (AOM) or chronic (COM) or serous (SOM), we used the following criteria:

1. AOM: diagnosis based on clinical data (fever and ear ache or irritability of sudden onset for less than one week) and the findings from the pneumatic otoscopy: hyperemia or opacity in an intact tympanic membrane, followed by its bulging or loss of mobility^6^, ^11^;
2. COM: the diagnosis was defined based on the presence of chronic inflammation of the middle ear and the mastoid mucosa, with a non-intact tympanic membrane and/or purulent otorrhea with a normal external auditory canal for more than six weeks^6^, ^12^, ^13^, ^14^;
3. SOM: diagnosis was defined based on the evidence of serous-purulent effusion from the middle ear with an intact tympanic membrane and without active infection^6^, ^15^, audiometry showing an air-bone gap and type B tympanometric curve.

On the same day of the first visit, a blood sample was collected in order to determine the serum count of CD4+ T lymphocytes.

### Statistics

The analysis of these 459 children was stratified according to age, and they were divided in two groups: from 0 to 5 years and 11 months, and from 6 to 12 years and 11 months. For each age range we compared and calculated OM prevalence, as well as CD4 + T cells among the patients under ART and those under HAART. We used the Pearson's chi-squared test and the Fisher test for categorical variables, and the non-parametric Mann-Whitney U test for the continuous variables. P values bellow 0.05 were considered statistically significant. Data was analyzed through the Statistical Package for Social Sciences (SPSS® for Windows 10.0, SPSS Inc., Chicago, IL) software.

This study was evaluated and approved by the Ethics in Research Committee of the Hospital.

## RESULTS

Among the 459 children, 152 (33.1%) had some type of otitis media. The COM was the most prevalent in both age groups, present in 65 (14.2%) children. Table 4 depicts the prevalence of chronic otitis media found and its distribution according to age and the use of HAART.

**Table 4:** Chronic otitis media prevalence among the children in the study, according to age and HAART use or not.

	Age range					
	0 to 5 years and 11 months			6 to 12 years and 11 months		
	HAART (n = 70)	ART	p	HAART	ART	p
Otite Média Crônica	5 (7,1%)	22 (20,8%)	0,02	20 (13,1%)	18 (13,8%)	0,86

HAART = Highly Active Antiretroviral Therapy; ART = Antiretroviral Therapy

At the age range between 6 and 12 years and 11 months, prevalence differences among the different types of otitis were not statistically significant among ART and HAART children. However, in the group of children with ages varying between 0 and 5 years and 11 months, the prevalence of chronic otitis media was significantly lower (p = 0.02). The relative risk of these children bellow 5 years and 11 months of age under the HAART regimen have chronic otitis media was of 0.4 (CI95%: 0.2 – 0.9) times the risk of those who were under ART.

Only one (0.2%) 7 year old child who was not using HAART had acute otitis media complicated with otomastoiditis. Her CD4+ lymphocyte count was of 397 × 10-9cel/L, and the use of intravenous antibiotics was efficient in curing her disease.

The average serum counts of CD4+ T lymphocytes for each age range are depicted on Tables 5 and 6. Among the children with ages varying between 0 and 5 years and 11 months, patients with COM had serum counts of CD4+ T cells of 150.5 ± 38 × 10-9 cell/L in average; below those without COM (p < 0.001).

**Table 5:** CD4+ T lymphocyte serum count according to the presence of chronic otitis media in children aged between 0 and 5 years and 11 months.

	0 to 5 years and 11 months (n = 176)	
	CD4+ (x10-9cel/L) count	p
Chronic Otitis Media		
Yes (n = 27)	658,2 ± 189,5	< 0,001
No (n = 149)	808,7 ± 179,9

**Table 6:** CD4+ T lymphocyte serum count according to the presence of chronic otitis media in children aged between 6 and 12 years and 11 months.

	6 to 12 years and 11 months (n = 283)	
	CD4+ (x10-9cel/L) Count	p
Chronic Otitis Media Yes (n = 38)	492,5 ± 163,6	0,7
No (n = 245)	505,4 ± 188,4	

Among the children between 6 and 12 years and 11 months, there was no statistically significant difference regarding lymphocyte counts when we compared children with COM with those without it.

In general, children with ages between 6 and 12 years and 11 months who had some type of otitis, had CD4+ counts of 75.5 38 × 10-9cel/L above the counts of other children (p = 0.002).

Children using HAART had average counts above that from those children using ART, regardless of age, and the differences were statistically significant (p < 0.001) – Table 7.

**Table 7:** CD4+ T Lymphocyte count according to age range and the use of HAART.

	Age Range			
	0 to 5 years and 11 months (n = 176)		6 to 12 years and 11 months (n = 283)	
	CD4+ (x109cel/L)	p	CD4+ (x109cel/L)	p
HAART (n = 223)	872,7 ± 158,1	< 0,001	539,1 ± 166,8	< 0,001
ART (n = 236)	728,1 ± 186,0		461,9 ± 197,1	

**HAART** = Highly Active Antiretroviral Therapy; **ART** = Antiretroviral Therapy

## DISCUSSION

Ear diseases are particularly common in HIV-infected children, and it is one of the major causes of referrals to the otorhinolaryngologist^16^. Aside from it, HIV-infected children have otitis media more frequently and with greater severity than their immunecompetent couterparts^8^. In our series, otitis media was the most prevalent otorhinolaryngological disease. On the other hand, reports in the otological infection literature in HIV-infected children under the HAART therapy are.

It is important to Record that all the patients included in the present study were receiving antiretroviral therapy, even if under heterogeneous therapeutic regimens. Since 1991, the Brazilian Government started to distribute Zidovudine free of charge for infected patients, and since 1996, by means of the Act 9313/96, 100% of infected patients have free access to antiretroviral therapy, including protease inhibitors.

The prevalence of chronic or acute otitis media in HIV-positive children in the literature varies. Chen et al. found a prevalence of 44% of recurrent otitis media in their series^16^. More recently, in South America, Bernaldez et al. found an incidence of 13.2% of suppurative chronic otitis media in HIV-infected children^14^. Now, Chandrasekhar et al. reported the presence of otorrhea in 5% of their HIV-infected patients^17^. Chaloryoo et al. found a general prevalence of otitis media in 18.4% in a group of 250 infected Thai children^18^. Singh et al. noticed a prevalence of 24% in a group of 107 children in London^19^. Hadfield et al., noticed a prevalence of 26% of acute otitis media and 7.5% of chronic otitis media in 66 children they followed for 8 years^20^. Such data is similar to what we found in the present study, in which we found a prevalence of 10.5% for AOM, 14.2% for COM and 8.5% for SOM.

As to the influence of antiretroviral therapy in the prevalence of otitis media in HIV-positive patients, Zuccotti et al. did not find statistically significant differences regarding the number of AOM episodes among children using or not antiretroviral therapy^21^.

Contrary to this, in our study, the use of HAART by HIV-positive children was associated to a lower prevalence of COM and greater prevalence of AOM, among children in the age range between 0 and 5 years and 11 months of age. As to SOM we did not notice any difference in prevalence among any age range studied, regardless of the use or not of the HAART therapy.

The relationship between clinical stage of the HIV infection, CD4+ lymphocyte count and otitis media was investigated by some authors. Chen et al. in their series have described a greater risk of otitis media infections as children progressed to more advanced clinical and immunological stages of the disease^16^. Makokha et al., in Kenya, showed the presence of acute otitis media in HIV-infected children associated to a low CD4+ T cell count^22^. Barnett et al. had already reported that children with low CD4+ lymphocyte counts had higher risk of presenting recurrent otitis media^9^. Such data is in disagreement with those we found, since in our series, children under the HAART therapy with greater CD4+ T lymphocyte counts had greater AOM prevalence.

Moreover, the COM pathogenesis is multifactorial: environmental factors, genetically determined factors and functional/anatomical characteristics of the Eustachian Tube, all play a role^13^. G (IgG) and A (IgA) immunoglobulins are important in the defense mechanisms against COM. There seems to be evidences of a regulatory role played by CD4+ T cells and interleukins 2 and 4 (IL-2 and IL-4) in the conversion of acute to chronic stages of OM, specially in cases of SOM^23^, ^24^.

On the other hand, local mucosa immunity plays an important role in the microecology of the nasopharynx bacterial flora, and the HIV-infected children seem to have a severe local immune disorder^11^.

In our study, the HAART therapy has allowed for a better cellular immunity due to a significant increase in the count of CD4+ lymphocytes in both age ranges, and such fact was also observed by other authors^4^, ^5^, ^25^, ^26^. This may have been one of the reasons that led the children under HAART, aged bellow 6 years to present lower COM incidence.

Another important point to be stressed in our study is the fact that we found only one child with complications accruing from otitis media (otomastoiditis). The literature states that otitis media tends to be more severe in the immunesupressed population with greater rates of complications, specially bacteremia^6^, ^9^, ^11^, ^16^. It is possible that this reduction seen in the incidence be secondary to the immunological improvement caused by HAART.

## FINAL COMMENTS

Since this is a transversal study, based on a single evaluation of patients, the major limitation we had is in establishing only associations between the data and not necessarily causality. Another possible limitation is that we do not control data related to HIV-1 RNA levels (viral burden), which were not available for all patients.

Thus, based on the data presented here, we conclude that the HAART therapy in HIV-infected children below 6 years of age was associated to a lower prevalence of chronic otitis media, probably due to an increase in their CD4+ T lymphocyte counts.

## References

[1] UNAIDS – Global HIV/AIDS 2004 report. Available at: http://www.unaids.org/EN/other/functionalities/search.asp. Accessed May 9, 2005

[2] Secretaria de Vigilância em saúde, Ministério da Saúde – Brasil (2004). Programa nacional de DST e AIDS (Brasil). Casos de AIDS (números e percentual) em indivíduos com 13 anos de idade ou menos, segundo categoria de exposição hierarquizada por sexo e ano de diagnóstico, 1980-2004. Boletim Epidemiológico AIDS e DST.

[3] Hoare S (2003). HIV infection in children – impact upon ENT doctors. Int J Pediatr Otorhinolaryngol.

[4] Gortmaker SL, Hughes M, Cervia J (2001). Effects of Combination Therapy Including Protease Inhibitors on Mortality Among Children and Adolescents Infected with HIV-1. N Eng J Med.

[5] Miziara ID, Valentini M, Romano FR, Miniti A (2002). Changing patterns of buccal manifestations in AIDS. Rev Laryngol Otol Rhinol.

[6] Shapiro NL, Novelli V (1998). Otitis media in children with vertically-acquired HIV infection: the Great Ormond Street Hospital experience. Int J Pediatr Otorhinolaryngol.

[7] Williams MA (1987). Head and neck findings in pediatric acquired immune deficiency syndrome. Laryngoscope.

[8] Rinaldo A, Brandwein MS, Devaney KO, Ferlito A (2003). AIDS-related otological lesions. Acta Otolaryngol.

[9] Barnett ED, Klein JO, Pelton SI, Lunginbuhl IM (1992). Otitis media in children born to human immunodeficiency virus infected mothers. Pediatr Infect Dis J.

[10] Caldwell MB, Oxtoby MJ, Simonds RJ, Lindegren ML, Rogers MF (1994). 1994 Revised Classification System for Human Immunodeficiency Virus Infection in Children Less Than 13 Years of Age. MMWR Morb Mortal Wkly Rep.

[11] Marchisio P, Principi N, Sorella S, Sala E, Tornaghi R (1996). Etiology of acute otitis media in Human Immunodeficiency Virus infected children. Pediatr Infect Dis J.

[12] Roland PS (2002). Chronic suppurative otitis media: a clinical overview. Ear Nose Throat J.

[13] Verhoeff M, van der Veen EL, Rovers MM, Sanders EAM, Schilder AGM (2005). Chronic suppurative otitis media: A review. Int J Pediatr Otorhinolaryngol.

[14] Bernaldez PC, Morales G, Hernandez CM (2005). Chronic suppurative otitis media in HIV-infected children: P140. Otolaryngol Head Neck Surg.

[15] Bluestone CD (1998). Epidemiology and pathogenesis of chronic suppurative otitis media: implications for prevention and treatment. Int J Pediatr Otorhinolaryngol.

[16] Chen AY, Ohlms LA, Stewart MG, Kline MW (1996). Otolaryngologic disease progression in children with human immunodeficiency virus infection. Arch Otolaryngol Head Neck Surg.

[17] Chandrasekhar SS, Connelly PE, Brambhatt SS, Shah CS, Kloser PC, Baredes S (2000). Otologic and audiologic evaluation of human immunodeficiency virus-infected patients. Am J Otolaryngol.

[18] Chaloryoo S, Chotpitayasunondh T, Chiengmai PN (1998). AIDS in ENT in children. Int J Pediatr Otorhinolaryngol.

[19] Singh A, Georgalas C, Patel N, Papesch M (2003). ENT presentations in children with HIV infection. Clin Otolaryngol.

[20] Hadfield PJ, Birchall MA, Novelli V, Bailey CM (1996). The ENT manifestations of HIV infection in children. Clin Otolaryngol Allied Sci.

[21] Zuccotti GV, D’Auria E, Torcoletti M, Lodi F, Bernardo L, Riva E (2001). Clinical and Pro-host Effects of Cefaclor in Prophylaxis of Recurrent Otitis Media in HIV-Infected Children. J Int Med Res.

[22] Makokha EP, Ogolla M, Orago AS (2003). CD4 T lymphocyte subsets and disease manifestation in children with and without HIV born to HIV-infected mothers. East Afr Med J.

[23] Smirnova MG, Birchall JP, Pearson JP (2005). Evidence of T-helper cell 2 cytokine regulation of chronic otitis media with effusion. Acta Otolaryngol.

[24] Skotnicka B, Stasiak-Barmuta A, Hassmann-Poznanska E, Kasprzycka E (2005). Lymphocyte subpopulations in middle ear effusions: flow cytometry analysis. Otol Neurotol.

[25] Granados JMS, Amador JTR, Miguel SF, Tomé MIG, Conejo PR, Vivas PF (2003). Impact of higly active antiretroviral therapy on the morbidity and mortality in Spanish human immunodeficiency virus-infected children. Pediatr Infect Dis J.

[26] Kline MW (2003). Human Immunodeficiency Virus Protease Inhibitors. Pediatr Infect Dis J.

